# Alcohol Consumption and Acute Coronary Syndrome: Epidemiology, Pathophysiology, and Clinical Perspectives

**DOI:** 10.3390/jcm15010299

**Published:** 2025-12-30

**Authors:** Panagiotis Iliakis, Eleftheria Stamou, Angeliki Vakka, Konstantina Ntalekou, Maria Kouremeti, Nikolaos Ktenopoulos, Paschalis Karakasis, Panagiotis Theofilis, Anna Pitsillidi, Athanasios Sakalidis, Kyriakos Dimitriadis, Christina Chrysochoou, Konstantinos Tsioufis

**Affiliations:** 1 First Department of Cardiology, School of Medicine, National and Kapodistrian University of Athens, Hippokration General Hospital, 11527 Athens, Greecemairhkou@gmail.com (M.K.); nikosktenop@gmail.com (N.K.); panos.theofilis@hotmail.com (P.T.); asakalidis@gmail.com (A.S.);; 2 Second Department of Cardiology, Hippokration General Hospital, 54642 Thessaloniki, Greece; 3 Department of OB/GYN, Rheinland Klinikum Dormagen, Dr. Geldmacher-Strasse 20, 41540 Dormagen, Germany

**Keywords:** alcohol, acute coronary syndrome, coronary artery disease, cardiovascular mortality, cardiovascular prevention

## Abstract

Alcohol consumption is a globally prevalent lifestyle factor with complex and sometimes paradoxical effects on cardiovascular health, particularly regarding acute coronary syndrome (ACS). Earlier epidemiological studies described a J-shaped relationship between alcohol consumption and ACS risk; however, emerging evidence has increasingly challenged the validity of this concept. Mendelian randomization studies, genetic data, and recent pooled analyses suggest that the apparent cardioprotective effects of light-to-moderate drinking are largely attributable to residual confounding, including abstainer bias and socioeconomic factors, rather than true causal mechanisms. In contrast, excessive alcohol intake is linked to increased oxidative stress, inflammation, hypertension, and prothrombotic states, all of which contribute to plaque instability and the precipitation of ACS. Additionally, acute heavy drinking episodes may induce coronary vasospasm and arrhythmias, further elevating ACS risk. Genetic factors, drinking patterns, and beverage types may also modulate the relationship between alcohol and ACS, indicating the need for personalized risk assessment. Understanding these complex interactions is essential for clinicians when counseling patients on alcohol consumption within the context of cardiovascular prevention. This review aims to delve into current evidence on the epidemiology and pathophysiology linking alcohol consumption with ACS, providing a nuanced perspective that balances potential protective effects with the significant risks associated with excessive alcohol use, as well as summarizing all medical societies’ recommendations regarding alcohol consumption and cardiovascular health.

## 1. Introduction

Alcohol consumption is a common social habit that carries both potential benefits and notable health risks, especially regarding cardiovascular health. Traditionally, its effects were considered biphasic: moderate drinking may provide some cardioprotective advantages, whereas excessive intake is associated with harmful outcomes [1]. However, this has been increasingly challenged, and the net impact of alcohol on cardiovascular health remains controversial based on conflicting findings across epidemiological studies. Alcohol has been associated with almost the whole spectrum of cardiovascular disease, such as hypertension, atrial fibrillation, and coronary artery disease with acute and chronic impact, while the underlying pathophysiology has not yet been clearly understood [2,3].

From a population health perspective, the global burden attributable to alcohol is substantial. According to recent data, alcohol is responsible for approximately 3 million deaths annually worldwide, and cardiovascular disease represents the largest share of alcohol-related deaths in the European Union [4]. Europe also has the highest rates of alcohol consumption globally, with approximately 290,000 deaths each year in the EU attributed to alcohol-related causes, including at least 50,000 CVD deaths. An alcohol intake of 100 g per week is associated with a linear increase in the risk of stroke, heart failure, fatal hypertensive disease, and fatal aortic aneurysm, with a borderline rise in the risk of coronary heart disease, compared with individuals consuming 0–25 g per week [5]. Recent high-quality pooled analyses have challenged the long-held belief that moderate drinking confers meaningful cardiovascular protection. In the context of acute coronary syndrome (ACS), the relationship between alcohol and risk is similarly complex. Light-to-moderate consumption has historically been associated with lower ACS incidence, partially attributed to favorable effects on lipid profiles, insulin sensitivity, endothelial function, and hemostatic balance [3,6]. Furthermore, even infrequent binge episodes have been associated with a two-fold increase in major adverse cardiovascular events after ACS, highlighting the importance of drinking patterns rather than average intake alone [7,8]. Moreover, heavy alcohol drinking is not only associated with higher ACS prevalence but with higher in- and out-of-hospital mortality [9,10].

While alcohol consumption and cardiovascular risk have been extensively addressed in high-profile scientific statements and general population studies, important knowledge gaps remain regarding the role of alcohol consumption in the setting of acute coronary syndromes. This manuscript was conducted as a narrative review. Studies were identified through targeted searches of PubMed and Scopus, focusing on studies published primarily over the last two decades, with particular emphasis on recent high-quality observational studies, Mendelian randomization analyses, pooled analyses, and guideline documents. Search terms included combinations of “alcohol consumption,” “drinking patterns,” “acute coronary syndrome,” “myocardial infarction,” and “cardiovascular risk”. This narrative review adopts an ACS-focused perspective, integrating recent epidemiological, mechanistic, and clinical data—including studies published after 2022—and it aims to explore the epidemiological associations between varying patterns of alcohol intake and the incidence of ACS while elucidating the underlying pathophysiological mechanisms, as well as the recommendations of all major medical societies towards ameliorating alcohol’s deleterious effect and promoting cardiovascular prevention.

## 2. Epidemiology of Alcohol Consumption and Acute Coronary Syndrome

### 2.1. Drinking Patterns and Epidemiology

According to the World Health Organization (WHO), global alcohol consumption declined slightly in 2019, while the highest levels were observed in Europe and the Region of the Americas [4]. Nevertheless, alcohol use disorders have increased in Africa, the Eastern Mediterranean, and Southeast Asian regions, making alcohol-related deaths an unresolved public health concern [4]. Specific drinking patterns have been established to describe alcohol consumption. This terminology is beneficial both in research and in everyday clinical practice, enabling assessment of short- and long-term health outcomes. Some studies quantify alcohol consumption in terms of drinks per unit of time or based on the concentration of ethanol ingested. In the United States of America (USA), a standard drink contains 14 g of pure alcohol. However, the alcohol concentration of a standard drink varies globally from 8 g (in Iceland and the United Kingdom) to 20 g (in Austria) [4]. The following patterns are commonly used [11,12]:

▪ Low-risk drinking: Defined by the National Institute on Alcohol Abuse and Alcoholism (NIAAA) of the USA as ≤3 drinks on any single day and ≤7 drinks per week for women. For men, it is defined as ≤4 drinks on any single day and ≤14 drinks per week.
▪ Moderate drinking: Defined as up to 1 or 2 drinks per day for women or men, respectively.
▪ Binge drinking: Achieving a blood alcohol concentration ≥ 0.08 g per deciliter (0.08%) or higher. The WHO defines this pattern as consuming at least 60 g of pure alcohol (>4–5 drinks) on one or more occasions in the last month. A synonym term for binge drinking is heavy episodic drinking (HED).
▪ Extreme binge drinking (high-intensity drinking): Exceeding ≥2 times the gender-specific binge drinking thresholds.
▪ Heavy drinking: Usually refers to binge drinking for >5 days over the last month, or in some studies, as a consumption of more than 2 drinks/day.
▪ Heavy continuous drinking (HCD): Denotes binge drinking regularly over the last year.

The current global average consumption of 27 g/day among drinkers highlights the magnitude of alcohol-related health risks [4]. Regarding binge drinking, the prevalence rates are 27% for females, 45% for males, and 38% for all adults [13,14,15]. In 2019, 3.6% of adults were identified as heavy continuous drinkers. Overall, males had a higher prevalence of HCD, at 6.7%, while females had a much lower prevalence, at 0.6% [12,16]. Moreover, an important methodological limitation in interpreting epidemiological studies on alcohol consumption and ACS risk is the lack of uniformity in the definition of a “standard drink” across regions, which ranges from approximately 8 g to 20 g of pure ethanol. This substantial variability hampers direct comparison between studies and complicates the aggregation of evidence across populations.

### 2.2. The J-Shaped Relationship Between Alcohol and ACS Risk and the Impact of Light-to-Moderate Drinking on ACS Incidence

According to the 2021 European Society of Cardiology (ESC) guidelines on cardiovascular disease prevention [17], alcohol intake should be restricted to a maximum of 100 g of pure alcohol per week. As a J-shaped relationship between alcohol and all-cause mortality or CV risk was often reported [3,6,7], light alcohol intake was thought to have a protective effect on all-cause and cardiovascular (CV) mortality. For instance, a large meta-analysis involving patients with cardiovascular disease (CVD) reported risk reduction at 6 g/day for cardiovascular events [3]. Moreover, it has been suggested that the lowest risk of mortality and recurrence of CV events is associated with low-to-moderate consumption in both male and female individuals [3]. The INTERHEART study, a multicenter case–control study of 12,461 individuals with myocardial infarction (MI), demonstrated that moderate alcohol use (<4 times/week) is associated with a lower risk of MI recurrence, though this protective effect was not uniform across geographic regions [8]. In line with ESC guidelines, moderate and non-binge-like alcohol consumption appeared to be safe in another prospective cohort study of 6557 patients presenting with ACS [6,17]. However, this U- or J-shaped relationship is refuted by recent evidence [5,18,19,20]. Pooled analyses of prospective studies indicate that alcohol consumption exhibits a curvilinear relationship with all-cause mortality, a linear association with coronary heart disease, and an inverse association with MI—particularly non-fatal events—suggesting that moderate intake may differentially influence the risk and severity of MI [5]. Estimates of reduced risk may have been overstated due to “abstainer bias” or the “sick quitter” phenomenon [5,21]. Other methodological limitations—including selection bias, failure to account for binge drinking patterns, and residual confounding—further complicate the interpretation of these findings [3]. Confounding factors, such as age, smoking, and also race/ethnicity (due to different drinking patterns and genetic backgrounds) should be taken into account [5]. Hence, the ‘French’ paradox is disproven, unmasking a more complex relationship between alcohol consumption and cardiovascular disease risk [22,23].

Historically, the J-shaped association between alcohol consumption and cardiovascular outcomes emerged primarily from observational studies, which are inherently vulnerable to residual confounding and selection bias. Factors such as the inclusion of former drinkers within abstainer groups, including the so-called “sick quitter” effect, the healthy drinker effect, and socioeconomic and lifestyle differences likely contributed to the apparent cardioprotective signal observed at low levels of alcohol intake. Additional bias may arise from misclassification of drinking status and changes in alcohol consumption over time, which are not adequately captured in many cohort studies. Importantly, while most observational studies have suggested a J-shaped association between alcohol intake and cardiovascular risk, Mendelian randomization studies and recent pooled analyses do not support a causal cardioprotective effect of moderate alcohol consumption, and randomized controlled trial evidence is lacking. This distinction between observational correlations and causality is critical for accurately interpreting the epidemiological evidence. Nevertheless, a modest inverse association may still persist in some large, well-adjusted cohorts, likely reflecting residual confounding by unmeasured health behaviors and socioeconomic factors, as well as exposure heterogeneity; any putative small biological benefit confined to a narrow subgroup would be difficult to disentangle from bias and is unlikely to outweigh population-level harms.

### 2.3. Consequences of Heavy and Binge Drinking on ACS Occurrence

Heavy or binge drinking is a hazardous behavior in many aspects, but additional data are required to reveal its cardiovascular impact. One large retrospective study with a 12-year clinical follow-up of ACS patients showed 40% greater risk of in-hospital mortality and major adverse cardiac events (MACEs) in heavy drinkers (compared with light drinkers), consistent with published results from previous studies [24,25]. Tessitore et al. also found that even infrequent episodes of binge drinking following an acute coronary syndrome (ACS) are associated with worse clinical outcomes, showing a two-fold increase in the risk of MACEs within one year of follow-up [6]. Interestingly, individuals reporting binge drinking showed an increased risk of acute MI in the subsequent 24 h, with a stronger effect among older participants, according to the INTERHEART study [8]. While moderate alcohol consumption exerts intricate cardiovascular effects, heavy drinking is consistently associated with adverse outcomes and should be avoided.

## 3. Pathophysiological Mechanisms Linking Alcohol and Acute Coronary Syndrome

The biological effects of alcohol on cardiovascular pathways are highly dose- and pattern-dependent, displaying a biphasic profile. Acute or high-dose exposure is predominantly associated with toxic mechanisms, including oxidative stress, endothelial dysfunction, platelet activation, and prothrombotic states, whereas some chronic low-dose exposures described in experimental and observational studies have been linked to more favorable effects on lipid metabolism and fibrinolysis. The following section discusses these mechanisms while acknowledging that the balance between potentially protective and deleterious effects is strongly influenced by dose, drinking pattern, and individual susceptibility.

It is of great importance that interpretation of experimental alcohol exposure also requires careful dose contextualization. Ethanol concentrations commonly used in in vitro studies (~10–50 mM) correspond to blood alcohol levels that humans can reach during moderate to heavy drinking, with ~25 mM approximating a BAC of ~0.08%. In endothelial models, low-to-moderate concentrations (~5–25 mM) support vascular homeostasis, whereas higher concentrations (≥50 mM) induce dysfunction and pro-atherosclerotic signaling [26].

### 3.1. Oxidative Stress and Inflammation

Oxidative stress and inflammation are important contributors to cardiovascular disease [27]. In a study with young and aging rats fed with a 5% (*v*/*v*) liquid alcohol diet, alcohol led to acceleration of cardiovascular aging, as it promotes oxidative stress, mitochondrial dysfunction, inflammation, and senescence in the heart and vasculature of both young and aging mice [28]. Also, vascular hypercontractility is present in mice after treatment with high doses of ethanol (20% *v*/*v*) for 12 weeks, and it is mediated by the renin–angiotensin–aldosterone system via the promotion of mitochondrial reactive oxygen species (ROS) production and mitochondrial dysfunction [29]. In human coronary artery endothelial cells (HCAECs), ROS production increases with both moderate (25 mM) and high (50 mM) doses of ethanol in a dose-dependent way. Similarly, treatment with acetaldehyde (10 μM and 25 μM), which is the primary metabolite of ethanol, leads to a dose-dependent increase in ROS generation [30]. However, the relationship between ethanol dose and endothelial inflammatory cytokine production (measured by interleukin-6 and interferon-γ) is j-shaped in HCAECs, with maximum inhibition by an ethanol dose of 25 mM. Thus, it is not known whether the response regarding ROS production due to low-dose ethanol treatment is beneficial or detrimental [30].

In humans, consumption of ethanol in 25% aqueous solution (*v*/*v*) at a concentration of 0.76 g/kg of body weight daily in two doses for 3 days results in excessive oxidative stress, as it increases lipid peroxidation, reduces antioxidant defenses, and alters the oxidation/antioxidant balance [31]. However, moderate consumption of wine (27 g of alcohol/day) for 8 weeks has antioxidant effects, since it decreases oxidized guanine species and protein carbonyl levels, while ethanol increases these markers of oxidative stress, according to an RCT which randomized patients with coronary heart disease to no alcohol, ethanol, or wine [32].

Moreover, chronic plus binge ethanol consumption in mice causes excessive myocardial oxidative stress and mitochondrial dysfunction, lipid accumulation in the cardiomyocytes, and cardiac contractile dysfunction, which are characteristics of alcohol-induced cardiomyopathy [33]. Patients with documented alcohol abuse have elevated plasma levels of tumor necrosis factor alpha (TNF-α), a molecule that induces inflammation [34]. Thus, high doses of ethanol, as well as binge ethanol consumption, lead to elevated oxidative stress and inflammation, whereas low doses of ethanol—especially in specific types of alcoholic beverages, such as wine—might have antioxidant effects.

### 3.2. Endothelial Dysfunction

Endothelial dysfunction has a main role in the pathogenesis of atherosclerosis [35]. Alcohol can cause alterations in the endothelial barrier integrity and concentrations of vasoactive substances produced by endothelium [36].

As opposed to acute toxic effects, chronic exposure of HCAECs to low doses of ethanol (25 mM) shows improved barrier integrity as assessed by increased transendothelial electrical resistance (TEER), inhibited cell adhesion molecule (CAM) mRNA expression, and inhibited monocyte chemotactic protein-1 (MCP-1) expression and monocyte adhesion. On the contrary, high-dose ethanol exposure (50 mM) leads to decreased TEER and increased CAM and MCP-1 expression in HCAECs, leading to elevated monocyte adhesion [30]. Similarly, the barrier integrity of human stem cell-derived brain microvascular endothelial cells is affected by alcohol in a concentration-dependent way, as 10 mM ethanol does not alter TEER, while 50 mM and 100 mM ethanol cause significant decreases in TEER of 16–20% and 44–50%, respectively [37].

Regarding vasoactive substances, red wine (in a dose of ethanol at 1 g/kg body weight) acutely increases the production of nitric oxide (NO) in healthy humans [38]. Furthermore, in rats, moderate alcohol consumption (7.5% *v*/*v*) for 8 weeks results in an increase in the plasma levels of NO, improvement in postischemic myocardial systolic and diastolic function, and attenuation of increased coronary vascular resistance, whereas higher alcohol consumption (18% of total calories) leads to impaired maximum vascular relaxation in rats [39]. In cultured human umbilical vein endothelial cells (HUVECs), ethanol at 10 and 50 mM/liter also significantly increases NO levels, while NO production is significantly reduced by higher concentrations of ethanol (100 and 150 mM/liter) [40]. Moreover, rats treated with high doses of ethanol (20% *v*/*v*) for 2 weeks have increased levels of ETA receptors in the heart and mesenteric artery and show an enhanced, ETA-dependent pressor response to endothelin-1 compared to controls [41].

The heterogeneous nature of acute coronary syndromes also raises the possibility that alcohol may differentially influence the risk of Type 1 versus Type 2 MI [42]. Mechanisms such as plaque instability, platelet activation, impaired fibrinolysis, and acute inflammation may preferentially contribute to Type 1 MI through promotion of plaque rupture and thrombosis, particularly in the setting of binge or heavy episodic drinking [43]. In contrast, alcohol-related hemodynamic effects—including acute hypertension, tachyarrhythmias, anemia, and coronary vasospasm—may predispose susceptible individuals to Type 2 MI by exacerbating myocardial oxygen supply–demand imbalance. Although direct comparative data remain limited, this distinction underscores the need to consider ACS phenotype when interpreting alcohol-related cardiovascular risk [44].

### 3.3. Inhibition of Fibrinolysis

According to an analysis of the Framingham Offspring Cohort, which consisted of 3223 adults free of cardiovascular disease, light-to-moderate alcohol consumption (3–7 drinks/week) is correlated with lower levels of fibrinogen, plasma viscosity, vWF, and factor VII, while higher levels of consumption are associated with reduced fibrinolytic activity, as shown by higher levels of plasminogen activator inhibitor antigen-1 (PAI-1) and tissue plasminogen activator (TPA) antigen [45]. It is noteworthy that adults that consume moderate amounts of wine have the lowest PAI-1 levels [16]. Moreover, patients with documented alcohol abuse have elevated levels of vWF, PAI-1, and fibrinogen [34].

Acute consumption of a moderate dose of ethanol (35 g) in male individuals causes a significant temporary fibrinolysis inhibition during the first 14 h after consumption, since it increases PAI-1, decreases tPA, increases plasma clot lysis time, and decreases global fibrinolytic capacity of whole blood [46]. In vitro addition of ethanol in blood samples from healthy subjects shows a strong impairment of fibrinolysis, even at an ethanol level of 1‰, suggesting that the aforementioned association could be streamed via inhibition of fibrinolysis [47].

Regarding platelets, acute consumption of alcohol at a dose equivalent to one drink (0.25 mL/kg) or two drinks (0.5 mL/kg) inhibit platelet aggregation in a dose-dependent manner [48]. Patients with severe acute alcohol intoxication have impaired primary hemostasis due to reduced platelet function, which reverses upon sobering [49]. According to the Framingham Offspring Study, chronic higher alcohol consumption is inversely associated with both platelet activation and aggregation, especially in men [50]. Data from the Framingham Heart Study of 3427 subjects also suggest that high alcohol consumption (≥8 drinks/week for females and ≥15 drinks/week for males) is associated with decreased platelet reactivity [51]. Thus, chronic light-to-moderate alcohol consumption may have pro-fibrinolytic effects, while high alcohol consumption and binge drinking can reduce both fibrinolytic activity and platelet function.

### 3.4. Alterations in Lipid Profile

HDL is a protective molecule against atherosclerosis that acts by removing excess cholesterol from foam cells [52]. The Dallas Heart Study, which consisted of 2919 participants, showed that increased intake of alcohol is associated with increased levels of markers of HDL metabolism [53]. Alcohol consumption causes a dose-dependent increase in HDL-C, possibly by increasing transport rates of the major HDL apolipoproteins apoA-I and -II [54]. Although heavy drinking and binge drinking have been associated with decreased risk of low HDL-C, they have also been correlated with increased risk of metabolic syndrome in Korean males due to increased risk of high blood pressure, elevated blood glucose, and TG levels [55]. Subjects with excessive alcohol consumption usually have extremely high levels of HDL-C [56]. However, this does not translate to CVD protection, as the relation between HDL-C levels and the risk for CVD seems to follow a U-shaped curve [57].

Taken together, the mechanistic effects of alcohol on pathways relevant to acute coronary syndromes appear to differ substantially according to both dose and pattern of exposure, as well as between acute and chronic intake. Acute alcohol exposure, particularly in the context of binge or heavy episodic drinking, is associated with oxidative stress, endothelial dysfunction, platelet activation, and impaired fibrinolysis, thereby promoting plaque instability and thrombosis [58]. In contrast, some chronic low-dose exposures observed in experimental and observational settings have been linked to favorable lipid profiles or endothelial signaling, which may partly explain the historically observed J-shaped associations in epidemiological studies [58,59]. However, a substantial proportion of this mechanistic evidence is derived from animal models and in vitro experiments. While certain pathways, such as endothelial dysfunction and inflammatory activation, have been corroborated in human observational and interventional studies, other molecular mechanisms remain largely experimental. These translational limitations should be considered when extrapolating mechanistic findings to clinical ACS risk [17,60].

## 4. Modifying Factors in the Alcohol–ACS Relationship

### 4.1. Role of Genetic Factors

The relationship between alcohol consumption and acute coronary syndrome (ACS) is complex. As discussed above, binge drinking is consistently associated with increased thrombotic risk and ACS events. Genetic variability substantially modifies this relationship by influencing alcohol metabolism, lipid regulation, oxidative stress, and inflammatory pathways [61]. Approximately 90% of ethanol degrades in the human liver in oxidative and nonoxidative metabolic pathways. The major enzymes involved are alcohol dehydrogenase (ADH) and aldehyde dehydrogenase (ALDH). Functionally relevant polymorphisms are found in genes encoding ADH1b and ADH1c, affecting ethanol degradation rates and alcohol intake in white populations [62,63]. Several studies indicate that ADH and ALDH2 variants may play an important role in the development of coronary artery disease (CAD) and MI [64,65]. A meta-analysis by Han et al. demonstrated that the ALDH2 rs671 polymorphism is significantly associated with increased CAD and MI risk [66]. Similarly, Hisamatsu et al. reported a higher prevalence of coronary artery calcification—a marker of subclinical CAD—in ALDH2 rs671 1 homozygotes compared with 2 homozygotes [67].

Moderate alcohol consumption is linked to lower levels of inflammatory and hemostatic markers, whereas heavy drinking elevates C-reactive protein and other inflammatory mediators [68,69]. Alcohol also disrupts vascular endothelial integrity, causing endothelial dysfunction and reducing nitric oxide bioavailability, which leads to increased vascular tone, hypertension, and exacerbation of CAD. Ethanol further enhances fibrinogen and von Willebrand factor levels, increasing platelet aggregation and thrombogenic potential. Additionally, heavy alcohol exposure induces oxidative stress and upregulates cytokines such as interleukin-6 and tumor necrosis factor-α, stimulating vascular smooth muscle proliferation and plaque instability [1,61,70]. Overall, the combination of genetic susceptibility and prolonged excessive alcohol consumption promotes atherogenesis and metabolic imbalance, thereby increasing the likelihood of acute coronary syndrome, stroke, and heart failure.

Genetic variability may partially explain interindividual differences in susceptibility to alcohol-related cardiovascular risk and should be considered in personalized counseling. Polymorphisms affecting alcohol metabolism, particularly in enzymes such as alcohol dehydrogenase and aldehyde dehydrogenase, influence acetaldehyde accumulation, drinking behavior, and downstream cardiometabolic effects, thereby modifying ACS risk at comparable levels of alcohol intake [59]. While routine genetic testing is not currently recommended in clinical practice, awareness of genetic predisposition may support a more cautious, individualized approach to risk stratification and counseling, especially in patients with premature or recurrent ACS.

Beyond individual susceptibility, genetic epidemiology has been instrumental in clarifying the causal relationship between alcohol consumption and cardiovascular risk. Mendelian randomization studies use genetic variants associated with alcohol metabolism or drinking behavior as proxies for lifelong alcohol exposure, thereby minimizing confounding and reverse causation inherent in observational studies. Across multiple analyses, genetically predicted higher alcohol consumption has not been associated with reduced myocardial infarction risk and has instead been linked to adverse cardiovascular traits, including elevated blood pressure. These findings directly challenge the causal interpretation of the J-shaped association observed in traditional epidemiological studies and support the conclusion that the apparent cardioprotective effects of moderate alcohol intake are largely non-causal [59,71].

### 4.2. Influence of Drinking Patterns and Beverage Types

As discussed in detail in Section 2, the historically described J-shaped association between alcohol consumption and cardiovascular risk is primarily derived from observational data and is subject to significant methodological limitations. In population-based studies, low-to-moderate alcohol consumption was linked to a lower risk of CAD compared with both abstinence and heavy drinking [72]. Overall, evidence suggests that all alcoholic beverages may offer some protection against coronary artery disease (CAD), with only minor differences between types. While beer and wine appear slightly more favorable, red wine does not seem significantly more protective than other wine types [73]. The reduced CAD risk is likely related to ethanol itself rather than non-alcoholic components like polyphenols [74]. Experimental studies indicate that red wine polyphenols, including flavonoids and resveratrol, may contribute additional antioxidant and anti-inflammatory effects that support cardiovascular health [22]. However, these associations are subject to residual confounding and have not been confirmed by randomized trials or Mendelian randomization analyses. Within this framework, drinking pattern and total ethanol exposure appear to be more relevant determinants of cardiovascular risk than beverage type.

### 4.3. Personalized Risk Assessment in Clinical Practice

Acute coronary syndrome (ACS) is a leading manifestation of cardiovascular disease and a major global health concern, responsible for nearly 17.8 million deaths annually. Its development is influenced by both non-modifiable factors—such as age, sex, and genetics—and modifiable factors, including hypertension, obesity, smoking, dyslipidemia, diabetes, inflammation, and alcohol consumption [75].

Alcohol consumption is a complex modifiable risk factor influenced by multiple patterns of use. The AUDIT4 tool helps assess drinking behavior by measuring frequency, quantity per drinking day, and binge episodes. Notably, gender differences have been described, with women experiencing harmful effects at lower doses (>7 units/week) than men (>14 units/week). The impact of binge drinking varies by age and environment, underscoring the need for individualized assessment [6,15]. According to the Global Burden of Disease 2020 study, individuals aged 15–39 years have no safe level of alcohol intake, as even minimal consumption increases overall health risk, primarily through injuries and acute events. Adults aged 40–64 years may experience limited cardioprotective effects at low-to-moderate levels (≈0.5 drinks/day). In those aged ≥65 years, very modest consumption may offer some benefit, but higher intake rapidly increases the risk of stroke and mortality [14]. Following ACS, moderate regular alcohol consumption does not appear to increase the incidence of major adverse cardiovascular events (MACEs); however, even infrequent binge episodes double the risk of MACEs within a year of the initial event [6]. Overall, individualized, age- and pattern-specific risk assessment in clinical practice of alcohol consumption is essential for effective secondary prevention in ACS patients.

In the context of personalized risk assessment, drinking patterns appear also to be as clinically relevant as total alcohol intake. Binge drinking and heavy episodic alcohol consumption are more frequently observed in younger patients and males and have been consistently associated with plaque instability, acute thrombosis, and a higher risk of recurrent ACS events, particularly in the early post-ACS period [17,58,60]. In contrast, older patients and women may exhibit increased susceptibility to lower levels of alcohol intake, with disproportionate effects on blood pressure, arrhythmogenicity, and bleeding risk [50,76,77,78]. These phenotype-specific differences underscore the importance of individualized counseling that considers age, sex, drinking pattern, and post-ACS status rather than relying solely on average alcohol consumption.

## 5. Clinical Implications and Recommendations for Cardiovascular Prevention

Overall, these findings challenge the long-held belief that moderate alcohol consumption provides universal cardiovascular benefits [17]. Taken together, the available evidence does not support recommending alcohol consumption for cardiovascular prevention, as the apparent protective associations observed in observational studies are not corroborated by causal inference approaches or randomized data.

Counseling patients on alcohol consumption should be individualized and based on the most recent evidence and guideline recommendations. The European Society of Cardiology (ESC) 2016 guidelines recommend that alcohol consumption should be limited to two glasses per day (20 g/day) for men and one glass per day (10 g/day) for women independently of the presence of cardiovascular disease (CVD) [79]. The 2018 ESC/ESH Guidelines for the Management of Arterial Hypertension recommend restricting alcohol consumption to less than 14 units per week for men and 8 units per week for women [80]. According to the 2023 European Society of Hypertension (ESH) Guidelines for the Management of Arterial Hypertension, adult men and women with elevated blood pressure or hypertension who consume three or more alcoholic drinks per day should be advised that reducing alcohol intake—ideally to near abstinence—will significantly lower blood pressure (Class I, Level B). Furthermore, alcohol should not be recommended for cardiovascular disease prevention, as the apparent protective effects of moderate drinking are likely confounded (Class III, Level B) [81]. According to the 2025 AHA Guidelines for the Prevention, Detection, Evaluation, and Management of High Blood Pressure in Adults, individuals, with or without hypertension, who consume alcohol should be counseled to achieve abstinence or, at least, to reduce intake to ≤1 drink/d for women and ≤2 drinks/d for men to prevent or treat elevated BP and hypertension (Class I, Level A). Individuals consuming six or more drinks daily who reduced their intake by half experienced an average blood pressure reduction of 5.5/4.0 mmHg, while those drinking three to five drinks per day saw smaller but still significant reductions [82].

The 2021 ESC Guidelines on Cardiovascular Disease Prevention in Clinical Practice recommend limiting alcohol consumption to a maximum of 100 g of pure alcohol per week (approximately 8–12 standard drinks, depending on national definitions), with the same limit for both men and women (Class I, Level B). Consumption above this threshold is associated with reduced life expectancy. In addition, limiting alcohol intake is advised to prevent atrial fibrillation and is particularly important for patients considered for oral anticoagulant therapy (Class IIa, Level B) [17]. The 2023 ESC Guidelines for the Management of Acute Coronary Syndromes highlight that abstainers have the lowest risk of cardiovascular events, that any alcohol intake increases blood pressure and body mass index, and that consuming more than 100 g of alcohol per week is linked to shorter life expectancy. Therefore, patients recovering from ACS are advised to adopt a comprehensive healthy lifestyle, including complete smoking cessation, adherence to a Mediterranean-style diet, restriction of alcohol intake, regular aerobic and resistance exercise, and reducing sedentary time [60]. Similarly, the 2023 AHA Guideline for the Management of Patients with Chronic Coronary Disease (CCD) recommends that alcohol intake be limited to ≤1 drink per day for women and ≤2 for men, as this may reduce cardiovascular and all-cause mortality (Class IIa, Level B). Importantly, patients with CCD should not be encouraged to consume alcohol for cardiovascular protection (Class III, Level B), since there is a lack of evidence from Mendelian studies or RCTs. Moreover, heavy alcohol use and binge drinking are associated with increased morbidity and mortality, elevated triglyceride levels, and potential drug–alcohol interactions with commonly used cardiovascular medications [83].

To sum up, research and international guideline recommendations strongly emphasize that alcohol should not be promoted for cardiovascular protection, particularly in individuals with or at risk for acute coronary syndromes. All of the above is summed up in Table 1. Although observational studies suggested potential benefits with light-to-moderate drinking, newer data indicate that abstinence offers the lowest cardiovascular risk and that even low-to-moderate intake may elevate blood pressure and body mass index. As a result, major European and American societies uniformly advocate minimizing alcohol consumption—preferably achieving abstinence, or at least maintaining intake within strict limits—to optimize cardiovascular health and improve long-term outcomes. It can be concluded that no level of alcohol consumption being considered unequivocally safe for cardiovascular health. This is in line with all aforementioned guidelines’ suggestions and current recommendations emphasizing on risk minimization and cardiovascular risk prevention, rather than defining a “safe” threshold of alcohol intake.

### Patient Counseling and Clinical Strategy After ACS

In patients with established acute coronary syndromes, alcohol counseling should be integrated into secondary prevention strategies and informed by the underlying pathophysiological mechanisms. Beyond acute coronary syndromes, growing evidence indicates that alcohol contributes to a broad spectrum of cardiovascular conditions through shared pathophysiological pathways, including hypertension, arrhythmogenesis, myocardial dysfunction, endothelial injury, and chronic kidney disease [84]. Given the association between acute alcohol intake and sympathetic activation, blood pressure elevation, endothelial dysfunction, arrhythmias, and prothrombotic states, avoidance of binge or heavy episodic drinking should be strongly emphasized [81,85,86]. Particular caution is warranted in patients with residual ischemia, reduced left ventricular function, or a history of atrial or ventricular arrhythmias, in whom alcohol may precipitate adverse events [87,88].

From a practical standpoint, clinicians should frame counseling around risk minimization rather than presumed cardioprotection, clearly communicating that alcohol should not be recommended for cardiovascular benefit. In patients receiving antithrombotic therapy, alcohol intake may further increase bleeding risk and interact with blood pressure control, reinforcing the rationale for abstinence or strict minimization. Given the integrated nature of cardiovascular health, clinical messaging may benefit from moving away from condition-specific risk framing toward a more holistic approach that recognizes alcohol as a cardiotoxic exposure and a contributor to cardiovascular multimorbidity. Personalized counseling should adopt a holistic approach that considers drinking patterns, comorbidities, medication interactions, behavior modification, and patient preferences is essential to optimize long-term outcomes after ACS [17,60,89].

## 6. Conclusions

Alcohol consumption is strongly associated with cardiovascular disease, cardiovascular mortality, and with the prevalence of acute coronary syndrome. It is important to note once again that there is scarce evidence for—and no randomized trials on—a protective effect of low-to-moderate alcohol consumption on cardiovascular health in general and on heart health in particular. It should also be highlighted that all results should be studied and discussed with caution. From a clinical and public health perspective, abstinence or minimization of alcohol consumption represents the safest approach, particularly for individuals at increased cardiovascular risk or following acute coronary syndrome. Importantly, alcohol consumption should not be recommended for cardiovascular prevention, as the apparent protective associations reported in observational studies are not supported by randomized trials or causal inference analyses.

A long-term randomized controlled trial assigning sustained alcohol consumption or abstinence to assess cardiovascular outcomes would face substantial ethical and practical limitations, including challenges in long-term adherence, contamination between groups, and the difficulty of justifying prolonged exposure to a substance with established health harms. In the absence of such trials, higher-quality evidence is most likely to emerge from complementary approaches such as Mendelian randomization, large prospective cohorts with repeated assessment of alcohol intake over time, target trial emulation using real-world healthcare data, and natural experiments arising from population-level policy changes.

Future research should focus on unresolved gaps, including the impact of specific drinking patterns, sex-specific susceptibility, and the effects of alcohol consumption in the post-ACS setting, to better inform individualized risk assessment and counseling. Patients’ non-adherence, as well as doctors’ inertia, are strongly associated with poor compliance with all aforementioned recommendations, and it is of high importance that both national and international medical societies should intensify efforts to promote guideline implementation through education, standardized protocols, and continuous quality improvement initiatives.

## Figures and Tables

**Table 1:** Scientific Societies Recommendations.

Scientific Society	Recommendation
AHA/ACC (2025) [82]	Adults with or without hypertension who currently consume alcohol should be advised to pursue a recommended goal of abstinence, or at least to reduce alcohol intake to ≤1 drink/d for women and ≤2 drinks/d for men to prevent or treat elevated BP and hypertension. Optimal goal is abstinence for all adults for best health outcomes; in patients who consume alcohol, aim for >50% reduction in daily intake to no more than 2 drinks/d in men or 1 drink/d in women.
ESH (2023) [81]	Adult men and women with elevated BP or hypertension who currently consume alcohol (≥3 drinks a/day) should be advised that reduction in alcohol intake close to abstinence will lower their BP. Alcohol should not be recommended for CVD prevention, as previous studies linking moderate consumption to lower CV risk are likely confounded.
ESC (2023) [60]	It is recommended that ACS patients adopt a healthy lifestyle, including Stopping all smoking of tobacco; Healthy diet (Mediterranean-style); Alcohol restriction; Regular aerobic physical activity and resistance exercise; Reduced sedentary time.
AHA (2023) [83]	In patients with CCD who consume alcohol, it is reasonable to limit alcohol intake (≤1 drink/d for women, ≤2 drinks/d for men) to reduce cardiovascular and all-cause death. Patients with CCD should not be advised to consume alcohol for the purpose of cardiovascular protection.
ESC (2021) [17]	It is recommended to restrict alcohol consumption to a maximum of 100 g per week.
ESC/ESH (2018) [80]	It is recommended to restrict alcohol consumption to Less than 14 units per week for men. Less than 8 units per week for women.
ESC (2016) [79]	Consumption of alcoholic beverages should be limited to 2 glasses per day (20 g/d of alcohol) for men and 1 glass per day (10 g/d of alcohol) for women.

## Data Availability

No new data were created or analyzed in this study. Data sharing is not applicable to this article.

## Abbreviations

The following abbreviations are used in this manuscript:

ACC	American College of Cardiology
ACS	Acute Coronary Syndrome
ADH	Alcohol Dehydrogenase
ADH1B	Alcohol Dehydrogenase 1B
ADH1C	Alcohol Dehydrogenase 1C
AHA	American Heart Association
ALDH	Aldehyde Dehydrogenase
ALDH2	Aldehyde Dehydrogenase 2
BMI	Body Mass Index
BP	Blood Pressure
CAD	Coronary Artery Disease
CCD	Chronic Coronary Disease
CI	Confidence Interval
CVD	Cardiovascular Disease
ESC	European Society of Cardiology
ESH	European Society of Hypertension
ETA	Endothelin Type A (receptor)
HCAEC	Human coronary artery endothelial cells
HCD	Heavy Continuous Drinking
HDL	High-Density Lipoprotein
HED	Heavy Episodic Drinking
HR	Hazard Ratio
HTN	Hypertension
IFN-γ	Interferon Gamma
IL-6	Interleukin-6
LDL	Low-Density Lipoprotein
MACE	Major Adverse Cardiovascular Events
MI	Myocardial Infarction
NO	Nitric Oxide
OR	Odds Ratio
PAI-1	Plasminogen Activator Inhibitor-1
RCT	Randomized Controlled Trial
ROS	Reactive Oxygen Species
RR	Relative Risk
tPA	Tissue Plasminogen Activator
TEER	Transendothelial Electrical Resistance
TG	Triglycerides
TNF-α	Tumor Necrosis Factor-Alpha
vWF	von Willebrand Factor
WHO	World Health Organization

## References

[1] Gupta S., Ahimsadasan N., Dalsania K., Jing L., Waraich H., Gupta K., Kaminska M., Balamane S., Garcia-Zamora S., Miranda-Arboleda A.F. (2026). Alcohol and Cardiovascular Disease. Am. J. Cardiol..

[2] Krittanawong C., Isath A., Rosenson R.S., Khawaja M., Wang Z., Fogg S.E., Virani S.S., Qi L., Cao Y., Long M.T. (2022). Alcohol Consumption and Cardiovascular Health. Am. J. Med..

[3] Costanzo S., Di Castelnuovo A., Donati M.B., Iacoviello L., de Gaetano G. (2010). Alcohol Consumption and Mortality in Patients With Cardiovascular Disease. J. Am. Coll. Cardiol..

[4] Shield K., Franklin A., Wettlaufer A., Sohi I., Bhulabhai M., Farkouh E.K., Radu I.-G., Kassam I., Munnery M., Remtulla R. (2025). National, Regional, and Global Statistics on Alcohol Consumption and Associated Burden of Disease 2000–20: A Modelling Study and Comparative Risk Assessment. Lancet Public Health.

[5] Wood A.M., Kaptoge S., Butterworth A.S., Willeit P., Warnakula S., Bolton T., Paige E., Paul D.S., Sweeting M., Burgess S. (2018). Risk Thresholds for Alcohol Consumption: Combined Analysis of Individual-Participant Data for 599 912 Current Drinkers in 83 Prospective Studies. Lancet.

[6] Tessitore E., Branca M., Heg D., Nanchen D., Auer R., Räber L., Klingenberg R., Windecker S., Lüscher T.F., Carballo S. (2024). Drinking Patterns of Alcohol and Risk of Major Adverse Cardiovascular Events after an Acute Coronary Syndrome. Eur. J. Prev. Cardiol..

[7] Xi B., Veeranki S.P., Zhao M., Ma C., Yan Y., Mi J. (2017). Relationship of Alcohol Consumption to All-Cause, Cardiovascular, and Cancer-Related Mortality in U.S. Adults. J. Am. Coll. Cardiol..

[8] Leong D.P., Smyth A., Teo K.K., McKee M., Rangarajan S., Pais P., Liu L., Anand S.S., Yusuf S. (2014). Patterns of Alcohol Consumption and Myocardial Infarction Risk. Circulation.

[9] Lindschou Hansen J., Tolstrup J.S., Jensen M.K., Grønbæk M., Tjønneland A., Schmidt E.B., Overvad K. (2011). Alcohol Intake and Risk of Acute Coronary Syndrome and Mortality in Men and Women with and without Hypertension. Eur. J. Epidemiol..

[10] Persampieri S., Schaffer A., De Martino L., Valli F., Lupi A. (2020). Alcohol Consumption and Acute Coronary Syndrome: “In Vino Veritas”. Cardiology.

[11] Alcohol Research: Current Reviews Editorial Staff (2018). Drinking Patterns and Their Definitions. Alcohol Res..

[12] Mukamal K.J., Conigrave K.M., Mittleman M.A., Camargo C.A., Stampfer M.J., Willett W.C., Rimm E.B. (2003). Roles of Drinking Pattern and Type of Alcohol Consumed in Coronary Heart Disease in Men. N. Engl. J. Med..

[13] Galán I., Fontán J., Ortiz C., López-Cuadrado T., Téllez-Plaza M., García-Esquinas E. (2024). Volume of Alcohol Intake, Heavy Episodic Drinking, and All-Cause Mortality in Spain: A Longitudinal Population-Based Study. Addict. Behav..

[14] Bryazka D., Reitsma M.B., Griswold M.G., Abate K.H., Abbafati C., Abbasi-Kangevari M., Abbasi-Kangevari Z., Abdoli A., Abdollahi M., Abdullah A.Y.M. (2022). Population-Level Risks of Alcohol Consumption by Amount, Geography, Age, Sex, and Year: A Systematic Analysis for the Global Burden of Disease Study 2020. Lancet.

[15] Wilsnack R.W., Wilsnack S.C., Gmel G., Kantor L.W. (2018). Gender Differences in Binge Drinking. Alcohol Res..

[16] Niu C., Dong J., Zhang P., Yang Q., Xue D., Liu B., Xiao D., Zhuang R., Li M., Zhang L. (2025). The Global Burden of Cardiovascular Disease Attributable to High Alcohol Use from 1990 to 2021: An Analysis for the Global Burden of Disease Study 2021. Front. Public Health.

[17] Visseren F.L.J., Mach F., Smulders Y.M., Carballo D., Koskinas K.C., Bäck M., Benetos A., Biffi A., Boavida J.-M., Capodanno D. (2021). 2021 ESC Guidelines on Cardiovascular Disease Prevention in Clinical Practice. Eur. Heart J..

[18] Stockwell T., Zhao J., Panwar S., Roemer A., Naimi T., Chikritzhs T. (2016). Do “Moderate” Drinkers Have Reduced Mortality Risk? A Systematic Review and Meta-Analysis of Alcohol Consumption and All-Cause Mortality. J. Stud. Alcohol Drugs.

[19] Biddinger K.J., Emdin C.A., Haas M.E., Wang M., Hindy G., Ellinor P.T., Kathiresan S., Khera A.V., Aragam K.G. (2022). Association of Habitual Alcohol Intake With Risk of Cardiovascular Disease. JAMA Netw. Open.

[20] Rodríguez E.C., Kallmeyer A., Tarín N., Cristóbal C., Huelmos A., Lázaro A.M.P., Aceña Á., Gutiérrez-Landaluce C., González-Lorenzo Ó., Lumpuy-Castillo J. (2025). Beyond Secondary Prevention Drugs: Added Benefit in Survival and Events of a Healthy Lifestyle in Patients after an Acute Coronary Syndrome. Am. J. Prev. Cardiol..

[21] Ding C., O’Neill D., Britton A. (2022). Trajectories of Alcohol Consumption in Relation to All-Cause Mortality in Patients with Cardiovascular Disease: A 35-Year Prospective Cohort Study. Addiction.

[22] Lippi G., Franchini M., Favaloro E., Targher G. (2010). Moderate Red Wine Consumption and Cardiovascular Disease Risk: Beyond the “French Paradox”. Semin. Thromb. Hemost..

[23] Lombardo M., Feraco A., Camajani E., Caprio M., Armani A. (2023). Health Effects of Red Wine Consumption: A Narrative Review of an Issue That Still Deserves Debate. Nutrients.

[24] Panagiotakos D.B., Pitsavos C., Chrysohoou C., Stefanadis C., Toutouzas P. (2001). Risk Stratification of Coronary Heart Disease Through Established and Emerging Lifestyle Factors in a Mediterranean Population: CARDIO2000 Epidemiological Study. Eur. J. Cardiovasc. Prev. Rehabil..

[25] Burazeri G., Kark J.D. (2011). Moderate Alcohol Intake, Though Not Regular Heavy Drinking, Is Protective for Acute Coronary Syndrome: A Population-Based, Case-Control Study in Southeast Europe. Ann. Epidemiol..

[26] Dolganiuc A., Szabo G. (2009). In Vitro and in Vivo Models of Acute Alcohol Exposure. World J. Gastroenterol..

[27] Myszko M., Bychowski J., Skrzydlewska E., Łuczaj W. (2025). The Dual Role of Oxidative Stress in Atherosclerosis and Coronary Artery Disease: Pathological Mechanisms and Diagnostic Potential. Antioxidants.

[28] Mukhopadhyay P., Yokus B., Paes-Leme B., Bátkai S., Ungvári Z., Haskó G., Pacher P. (2025). Chronic Alcohol Consumption Accelerates Cardiovascular Aging and Decreases Cardiovascular Reserve Capacity. Geroscience.

[29] Awata W.M.C., Alves J.V., Costa R.M., Bruder-Nascimento A., Singh S., Barbosa G.S., Tirapelli C.R., Bruder-Nascimento T. (2023). Vascular Injury Associated with Ethanol Intake Is Driven by AT1 Receptor and Mitochondrial Dysfunction. Biomed. Pharmacother..

[30] Rajendran N.K., Liu W., Cahill P.A., Redmond E.M. (2023). Alcohol and Vascular Endothelial Function: Biphasic Effect Highlights the Importance of Dose. Alcohol. Clin. Exp. Res..

[31] Metro D., Corallo F., Fedele F., Buda M., Manasseri L., Buono L.V., Quartarone A., Bonanno L. (2022). Effects of Alcohol Consumption on Oxidative Stress in a Sample of Patients Recruited in a Dietary Center in a Southern University Hospital: A Retrospective Study. Medicina.

[32] Choleva M., Argyrou C., Detopoulou M., Donta M.-E., Gerogianni A., Moustou E., Papaemmanouil A., Skitsa C., Kolovou G., Kalogeropoulos P. (2022). Effect of Moderate Wine Consumption on Oxidative Stress Markers in Coronary Heart Disease Patients. Nutrients.

[33] Matyas C., Varga Z.V., Mukhopadhyay P., Paloczi J., Lajtos T., Erdelyi K., Nemeth B.T., Nan M., Hasko G., Gao B. (2016). Chronic plus Binge Ethanol Feeding Induces Myocardial Oxidative Stress, Mitochondrial and Cardiovascular Dysfunction, and Steatosis. Am. J. Physiol. Heart Circ. Physiol..

[34] Pacia K., Kaźnica-Wiatr M., Hat M., Pragnący K., Noga M., Podolec P., Olszowska M. (2024). Effect of Alcohol Abuse on Selected Markers of Inflammation, Hemostasis, and Endothelial Function. Cardiol. J..

[35] Tsalamandris S., Koliastasis L., Miliou A., Oikonomou E., Papageorgiou N., Antonopoulos A., Hatzis G., Mourouzis K., Vogiatzi G., Siasos G. (2025). Endothelial Function and Pro-Inflammatory Cytokines as Prognostic Markers in Acute Coronary Syndromes. Diagnostics.

[36] Gusti Y., Liu W., Athar F., Cahill P.A., Redmond E.M. (2025). Endothelial Homeostasis Under the Influence of Alcohol—Relevance to Atherosclerotic Cardiovascular Disease. Nutrients.

[37] Bell K.T., Hughes J.M., Borman W.A., Stoffel R.D., Canfield S.G. (2025). Alcohol Diminishes Barrier Integrity in Human Stem Cell-Derived Brain Microvascular Endothelial Cells: Role of Reactive Oxygen Species. Alcohol.

[38] Matsuo S., Nakamura Y., Takahashi M., Ouchi Y., Hosoda K., Nozawa M., Kinoshita M. (2001). Effect of Red Wine and Ethanol on Production of Nitric Oxide in Healthy Subjects. Am. J. Cardiol..

[39] Abou-agag L.H., Khoo N.K., Binsack R., White C.R., Darley-Usmar V., Grenett H.E., Booyse F.M., Digerness S.B., Zhou F., Parks D.A. (2005). Evidence of Cardiovascular Protection by Moderate Alcohol: Role of Nitric Oxide. Free Radic. Biol. Med..

[40] Kuhlmann C.R.W., Li F., Lüdders D.W., Schaefer C.A., Most A.K., Backenköhler U., Neumann T., Tillmanns H., Waldecker B., Erdogan A. (2004). Dose-Dependent Activation of Ca^2+^-Activated K^+^ Channels by Ethanol Contributes to Improved Endothelial Cell Functions. Alcohol. Clin. Exp. Res..

[41] Tirapelli C.R., Legros E., Brochu I., Honoré J., Lanchote V.L., Uyemura S.A., De Oliveira A.M., D’Orléans-Juste P. (2008). Chronic Ethanol Intake Modulates Vascular Levels of Endothelin-1 Receptor and Enhances the Pressor Response to Endothelin-1 in Anaesthetized Rats. Br. J. Pharmacol..

[42] Thygesen K., Alpert J.S., Jaffe A.S., Chaitman B.R., Bax J.J., Morrow D.A., White H.D., Executive Group on behalf of the Joint European Society of Cardiology (ESC)/American College of Cardiology (ACC)/American Heart Association (AHA)/World Heart Federation (WHF) Task Force for the Universal Definition of Myocardial Infarction (2018). Fourth Universal Definition of Myocardial Infarction (2018). Circulation.

[43] Flesch M., Rosenkranz S., Erdmann E., Böhm M. (2001). Alcohol and the Risk of Myocardial Infarction. Basic Res. Cardiol..

[44] Mostofsky E., Chahal H.S., Mukamal K.J., Rimm E.B., Mittleman M.A. (2016). Alcohol and Immediate Risk of Cardiovascular Events: A Systematic Review and Dose-Response Meta-Analysis. Circulation.

[45] Mukamal K.J., Jadhav P.P., D’Agostino R.B., Massaro J.M., Mittleman M.A., Lipinska I., Sutherland P.A., Matheney T., Levy D., Wilson P.W.F. (2001). Alcohol Consumption and Hemostatic Factors. Circulation.

[46] Pieters M., Vorster H., Jerling J., Venter C., Kotze R., Bornman E., Malfliet J., Rijken D. (2010). The Effect of Ethanol and Its Metabolism on Fibrinolysis. Thromb. Haemost..

[47] Engström M., Schött U., Reinstrup P. (2006). Ethanol Impairs Coagulation and Fibrinolysis in Whole Blood: A Study Performed with Rotational Thromboelastometry. Blood Coagul. Fibrinolysis.

[48] Zhang Q., Das K., Siddiqui S., Myers A.K. (2000). Effects of Acute, Moderate Ethanol Consumption on Human Platelet Aggregation in Platelet-Rich Plasma and Whole Blood. Alcohol. Clin. Exp. Res..

[49] Schmölders R.S., Hoffmann T., Hermsen D., Bernhard M., Boege F., Lau M., Hartung B. (2025). Evaluation of Alcohol Intoxication on Primary and Secondary Haemostasis—Results from Comprehensive Coagulation Testing. Int. J. Legal Med..

[50] Mukamal K.J., Massaro J.M., Ault K.A., Mittleman M.A., Sutherland P.A., Lipinska I., Levy D., D’Agostino R.B., Tofler G.H. (2005). Alcohol Consumption and Platelet Activation and Aggregation Among Women and Men: The Framingham Offspring Study. Alcohol. Clin. Exp. Res..

[51] Pashek R.E., Nkambule B.B., Chan M.V., Thibord F., Lachapelle A.R., Cunha J., Chen M.-H., Johnson A.D. (2023). Alcohol Intake Including Wine Drinking Is Associated with Decreased Platelet Reactivity in a Large Population Sample. Int. J. Epidemiol..

[52] Barter P. (2005). The Role of HDL-Cholesterol in Preventing Atherosclerotic Disease. Eur. Heart J. Suppl..

[53] Badia R.R., Pradhan R.V., Ayers C.R., Chandra A., Rohatgi A. (2023). The Relationship of Alcohol Consumption and HDL Metabolism in the Multiethnic Dallas Heart Study. J. Clin. Lipidol..

[54] De Oliveira e Silva E.R., Foster D., McGee Harper M., Seidman C.E., Smith J.D., Breslow J.L., Brinton E.A. (2000). Alcohol Consumption Raises HDL Cholesterol Levels by Increasing the Transport Rate of Apolipoproteins A-I and A-II. Circulation.

[55] Oh J.E. (2018). Relationship between Heavy Drinking, Binge Drinking, and Metabolic Syndrome in Obese and Non-Obese Korean Male Adults. Nutr. Res. Pract..

[56] Enriquez-Martinez O.G., Silva Pereira T.S., Mill J.G., da Fonseca M.d.J.M., Molina M.d.C.B., Griep R.H. (2023). Excessive Consumption of Alcoholic Beverages and Extremely High Levels of High-Density Lipoprotein Cholesterol (HALP) in the ELSA-Brasil Cohort Baseline. Nutrients.

[57] Zvintzou E., Karampela D.S., Vakka A., Xepapadaki E., Karavia E.A., Hatziri A., Giannopoulou P.C., Kypreos K.E. (2021). High Density Lipoprotein in Atherosclerosis and Coronary Heart Disease: Where Do We Stand Today?. Vascul. Pharmacol..

[58] Roerecke M., Rehm J. (2010). Irregular Heavy Drinking Occasions and Risk of Ischemic Heart Disease: A Systematic Review and Meta-Analysis. Am. J. Epidemiol..

[59] Holmes M.V., Dale C.E., Zuccolo L., Silverwood R.J., Guo Y., Ye Z., Prieto-Merino D., Dehghan A., Trompet S., Wong A. (2014). Association between Alcohol and Cardiovascular Disease: Mendelian Randomisation Analysis Based on Individual Participant Data. BMJ.

[60] Byrne R.A., Rossello X., Coughlan J.J., Barbato E., Berry C., Chieffo A., Claeys M.J., Dan G.-A., Dweck M.R., Galbraith M. (2023). 2023 ESC Guidelines for the Management of Acute Coronary Syndromes. Eur. Heart J..

[61] Piano M.R., Marcus G.M., Aycock D.M., Buckman J., Hwang C.-L., Larsson S.C., Mukamal K.J., Roerecke M. (2025). Alcohol Use and Cardiovascular Disease: A Scientific Statement From the American Heart Association. Circulation.

[62] Marussin A.V., Stepanov V.A., Spiridonova M.G., Khar’kov V.A., Pel’s J.R., Puzyrev V.P. (2007). Association Analysis of Alcohol Metabolizing Enzymes ADH1B, ADH7, CYP2E1 Gene Polymorphism with Risk for Coronary Atherosclerosis. Russ. J. Genet..

[63] Husemoen L.L.N., Jørgensen T., Borch-Johnsen K., Hansen T., Pedersen O., Linneberg A. (2010). The Association of Alcohol and Alcohol Metabolizing Gene Variants with Diabetes and Coronary Heart Disease Risk Factors in a White Population. PLoS ONE.

[64] Jo S.A., Kim E.-K., Park M.H., Han C., Park H.-Y., Jang Y., Song B.J., Jo I. (2007). A Glu487Lys Polymorphism in the Gene for Mitochondrial Aldehyde Dehydrogenase 2 Is Associated with Myocardial Infarction in Elderly Korean Men. Clin. Chim. Acta.

[65] Zhu L.-P., Yin W.-L., Peng L., Zhou X.-H., Zhou P., Xuan S.-X., Luo Y., Chen C., Cheng B., Lin J.-D. (2021). Association of Aldehyde Dehydrogenase 2 Gene Polymorphism with Myocardial Infarction. J. Inflamm. Res..

[66] Han H., Wang H., Yin Z., Jiang H., Fang M., Han J. (2013). Association of Genetic Polymorphisms in ADH and ALDH2 with Risk of Coronary Artery Disease and Myocardial Infarction: A Meta-Analysis. Gene.

[67] Hisamatsu T., Miura K., Tabara Y., Sawayama Y., Kadowaki T., Kadota A., Torii S., Kondo K., Yano Y., Fujiyoshi A. (2022). Alcohol Consumption and Subclinical and Clinical Coronary Heart Disease: A Mendelian Randomization Analysis. Eur. J. Prev. Cardiol..

[68] Moura H.F., Hansen F., Galland F., Silvelo D., Rebelatto F.P., Ornell F., Massuda R., Scherer J.N., Schuch F., Kessler F.H. (2022). Inflammatory Cytokines and Alcohol Use Disorder: Systematic Review and Meta-Analysis. Braz. J. Psychiatry.

[69] Imhof A., Froehlich M., Brenner H., Boeing H., Pepys M.B., Koenig W. (2001). Effect of Alcohol Consumption on Systemic Markers of Inflammation. Lancet.

[70] Tanaka A., Cui R., Kitamura A., Liu K., Imano H., Yamagishi K., Kiyama M., Okada T., Iso H., CIRCS Investigators (2016). Heavy Alcohol Consumption Is Associated with Impaired Endothelial Function. J. Atheroscler. Thromb..

[71] Millwood I.Y., Walters R.G., Mei X.W., Guo Y., Yang L., Bian Z., Bennett D.A., Chen Y., Dong C., Hu R. (2019). Conventional and Genetic Evidence on Alcohol and Vascular Disease Aetiology: A Prospective Study of 500 000 Men and Women in China. Lancet.

[72] Patel A., Figueredo V.M. (2023). Alcohol and Cardiovascular Disease: Helpful or Hurtful. Rev. Cardiovasc. Med..

[73] Klatsky A.L., Armstrong M.A., Friedman G.D. (1997). Red Wine, White Wine, Liquor, Beer, and Risk for Coronary Artery Disease Hospitalization. Am. J. Cardiol..

[74] Song R.J., Nguyen X.-M.T., Quaden R., Ho Y.-L., Justice A.C., Gagnon D.R., Cho K., O’Donnell C.J., Concato J., Gaziano J.M. (2018). Alcohol Consumption and Risk of Coronary Artery Disease (from the Million Veteran Program). Am. J. Cardiol..

[75] Jung E., Ryu H.H., Cho Y.J., Chun B.J. (2024). Interactions Between Depression, Alcohol Intake, and Smoking on the Risk of Acute Coronary Syndrome. Psychiatry Investig..

[76] Mukamal K.J., Chen C.M., Rao S.R., Breslow R.A. (2010). Alcohol Consumption and Cardiovascular Mortality Among U.S. Adults, 1987 to 2002. J. Am. Coll. Cardiol..

[77] Chrysohoou C., Iliakis P., Pitsillidi A., Manta E., Barkas F., Liberopoulos E., Sfikakis P.P., Pitsavos C., Tsioufis C., Panagiotakos D. (2025). Twenty-Year Cardiovascular Disease Incidence in Menopausal Women: Insights from the ATTICA Study. Climacteric.

[78] Camafort M., Kasiakogias A., Agabiti-Rosei E., Masi S., Iliakis P., Benetos A., Jeong J.-O., Lee H.-Y., Muiesan M.L., Sudano I. (2025). Hypertensive Heart Disease in Older Patients: Considerations for Clinical Practice. Eur. J. Intern. Med..

[79] Piepoli M.F., Hoes A.W., Agewall S., Albus C., Brotons C., Catapano A.L., Cooney M.-T., Corrà U., Cosyns B., Deaton C. (2016). 2016 European Guidelines on Cardiovascular Disease Prevention in Clinical Practice. Eur. Heart J..

[80] Williams B., Mancia G., Spiering W., Agabiti Rosei E., Azizi M., Burnier M., Clement D.L., Coca A., de Simone G., Dominiczak A. (2018). 2018 ESC/ESH Guidelines for the Management of Arterial Hypertension. Eur. Heart J..

[81] Mancia G., Kreutz R., Brunström M., Burnier M., Grassi G., Januszewicz A., Muiesan M.L., Tsioufis K., Agabiti-Rosei E., Algharably E.A.E. (2023). 2023 ESH Guidelines for the Management of Arterial Hypertension The Task Force for the Management of Arterial Hypertension of the European Society of Hypertension. J. Hypertens..

[82] Jones D.W., Ferdinand K.C., Taler S.J., Johnson H.M., Shimbo D., Abdalla M., Altieri M.M., Bansal N., Bello N.A., Writing Committee Members (2025). 2025 AHA/ACC/AANP/AAPA/ABC/ACCP/ACPM/AGS/AMA/ASPC/NMA/PCNA/SGIM Guideline for the Prevention, Detection, Evaluation and Management of High Blood Pressure in Adults: A Report of the American College of Cardiology/American Heart Association Joint Committee on Clinical Practice Guidelines. Hypertension.

[83] Virani S.S., Newby L.K., Arnold S.V., Bittner V., Brewer L.C., Demeter S.H., Dixon D.L., Fearon W.F., Hess B., Johnson H.M. (2023). 2023 AHA/ACC/ACCP/ASPC/NLA/PCNA Guideline for the Management of Patients with Chronic Coronary Disease: A Report of the American Heart Association/American College of Cardiology Joint Committee on Clinical Practice Guidelines. Circulation.

[84] Stambolliu E., Iliakis P., Tsioufis K., Damianaki A. (2025). Managing Hypertension in Chronic Kidney Disease: The Role of Diet and Guideline Recommendations. J. Clin. Med..

[85] Voskoboinik A., Prabhu S., Ling L.-H., Kalman J.M., Kistler P.M. (2016). Alcohol and Atrial Fibrillation: A Sobering Review. J. Am. Coll. Cardiol..

[86] O’Donnell M.J., Xavier D., Liu L., Zhang H., Chin S.L., Rao-Melacini P., Rangarajan S., Islam S., Pais P., McQueen M.J. (2010). Risk Factors for Ischaemic and Intracerebral Haemorrhagic Stroke in 22 Countries (the INTERSTROKE Study): A Case-Control Study. Lancet.

[87] Tsiachris D., Papakonstantinou P.E., Doundoulakis I., Tsioufis P., Botis M., Dimitriadis K., Leontsinis I., Kordalis A., Antoniou C.-K., Mantzouranis E. (2023). Anticoagulation Status and Left Atrial Appendage Occlusion Indications in Hospitalized Cardiology Patients with Atrial Fibrillation: A Hellenic Cardiorenal Morbidity Snapshot (HECMOS) Sub-Study. Medicina.

[88] Dimitriadis K., Soulaidopoulos S., Doundoulakis I., Iliakis P., Tsiachris D., Tsioufis P., Beneki E., Sakalidis A., Pagkalidou E., Tsiamis E. (2023). A Network Meta-Analysis of the Antithrombotic Strategies in Patients with Atrial Fibrillation and Percutaneous Coronary Interventions: Focus on Bleeding. Hell. J. Cardiol..

[89] Fragoulis C., Spanorriga M.-K., Bega I., Prentakis A., Kontogianni E., Tsioufis P.-A., Palkopoulou M., Ntalakouras J., Iliakis P., Leontsinis I. (2025). Behavioural Cardiology: A Review on an Expanding Field of Cardiology-Holistic Approach. J. Pers. Med..

